# Case report: Consecutive hyperbaric oxygen therapy for delayed post-hypoxic leukoencephalopathy resulting from CHANTER syndrome caused by opioid intoxication

**DOI:** 10.3389/fmed.2024.1364038

**Published:** 2024-04-17

**Authors:** Naoto Jingami, Kosai Cho, Takayuki Nitta, Miwa Takatani, Katsuya Kobayashi, Ryo Takenaka, Naoko Sugita, Shigeru Ohtsuru

**Affiliations:** ^1^Department of Primary Care and Emergency Medicine, Kyoto University Graduate School of Medicine, Kyoto, Japan; ^2^Hyperbaric Oxygen Therapy Center, Kyoto University Hospital, Kyoto, Japan; ^3^Department of Neurology, Kyoto University Graduate School of Medicine, Kyoto, Japan; ^4^Department of Psychiatry, Kyoto University Graduate School of Medicine, Kyoto, Japan

**Keywords:** hyperbaric oxygen therapy, delayed post-hypoxic leukoencephalopathy, delayed neurological sequelae, opioid intoxication, carbon monoxide

## Abstract

Delayed post-hypoxic leukoencephalopathy (DPHL) is a poorly recognized syndrome characterized by neuropsychiatric symptoms following recovery from an acute hypoxic episode. Although most cases are related to carbon monoxide poisoning, some have been linked to excessive opioid use. Opioid intoxication has recently become known for manifesting the characteristic imaging findings involving cerebellar, hippocampal, and basal nuclei transient edema with restricted diffusion (CHANTER) syndrome. Herein, we present a patient with severe disturbances in consciousness who was initially diagnosed with CO poisoning but was later found to have taken excessive tramadol. Magnetic resonance imaging (MRI) in the acute phase revealed abnormal intensities in the bilateral globus pallidus and the cerebellum, indicative of CHANTER syndrome. After intensive care, his level of consciousness was restored. However, around the 3rd week after hospitalization, his consciousness gradually deteriorated and he developed severe neurological symptoms. Another MRI on day 25 revealed a new diffuse white matter abnormality; DPHL was suspected. Cerebrospinal fluid collected on day 28 revealed significantly elevated myelin basic protein levels. Although it was challenging to decide on a treatment plan, hyperbaric oxygen (HBO) therapy trials were initiated on day 58; the patient's condition improved after a series of HBO sessions. MRI revealed gradual shrinkage of the white matter abnormality. A total of 63 consecutive HBO sessions were performed, leading to the successful resolution of the serious neurological symptoms. While the effectiveness of HBO therapy for DPHL remains inconclusive, especially in opioid-related cases, this patient made a remarkable recovery, likely due to the therapeutic effect of improved cerebral blood flow and oxygenation.

## 1 Introduction

Delayed post-hypoxic leukoencephalopathy (DPHL) is an under-recognized syndrome that manifests neuropsychiatric symptoms after recovery from an acute hypoxic episode (1). The course of the disease may improve or lead to permanent disability. Currently, no effective treatment is available, and supportive care and rehabilitation are the primary focuses. Most cases of DPHL are associated with carbon monoxide (CO) poisoning. However, cases associated with opioid overdoses have also been reported (2).

Opioid abuse disturbs consciousness and brain function. Recently, a clinicoradiological entity related to opioid intoxication involving cerebellar, hippocampal, and basal nuclei transient edema with restricted diffusion has been reported and ascribed the term CHANTER syndrome (3).

Our facility has a multi-person hyperbaric oxygen (HBO) therapy chamber, and we routinely perform HBO therapy on patients, including critically ill patients requiring tracheal intubation. HBO is known for improving various pathological conditions by enhancing the hypoxic environment in the body. HBO is also performed for DPHL after CO poisoning and is sometimes effective. However, its efficacy remains inconclusive. Therefore, HBO is still performed on a case-by-case basis.

Herein, we present the case of a patient who was diagnosed with CHANTER syndrome due to an opioid overdose, followed by DPHL, and treated with consecutive HBO.

## 2 Case description

**Figure 1:**
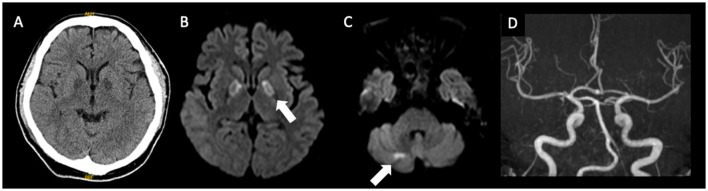
Imaging findings at the initial examination, **(A)**, CT; **(B)**, **(C)**: MRI; **(D)**, MRA. Bilateral involvement of the globus pallidus and abnormalities in the right cerebellar hemisphere are evident. No major vessel stenosis is observed. CT, computed tomography; MRI, magnetic resonance imaging; MRA, magnetic resonance angiography.

A 47-year-old male patient was admitted to our hospital due to severe disturbances in consciousness (Glasgow Coma Scale [GCS] score of 3). Hypoxemia due to impaired consciousness, which required respiratory management with intubation, was observed. Initial magnetic resonance imaging (MRI) revealed abnormal intensities in the bilateral globus pallidus and cerebellum (Figure 1). Although the carboxyhemoglobin level was as low as 1.2 %, CO poisoning was initially suspected based on characteristic MRI findings.

Daily HBO (2.8 ATA, 60 min) therapy was initiated based on the tentative diagnosis of CO poisoning. However, his family later found numerous empty tramadol-acetaminophen packing sheets (tramadol/acetaminophen dose equivalent: 1,575 mg/13,650 mg), which led to the diagnosis of tramadol intoxication. Subsequently, the serum acetaminophen concentration, measured over 15 h post-drug ingestion during transport, was notably high at 10.5 ug/ml, indicating a pharmacokinetic overdose. Tramadol was prescribed by a local physician for lumbago. HBO therapy was performed for 3 days. In addition, acetylcysteine via the gastric tube and intravenous naloxone were administered. His level of consciousness recovered to a GCS score of 14; the patient was extubated.

The patient was hospitalized, wherein rehabilitation was continued. However, his consciousness gradually deteriorated on the 20th day of hospitalization. Another MRI on day 25 revealed a new diffuse white matter lesion, and DPHL was suspected. Cerebrospinal fluid collected on day 32 revealed that the myelin basic protein levels were significantly elevated to 135.5 pg/mL. The patient was bedridden, unable to communicate, and had difficulty in ingesting food. Therefore, gastrostomy was considered.

**Figure 2:**
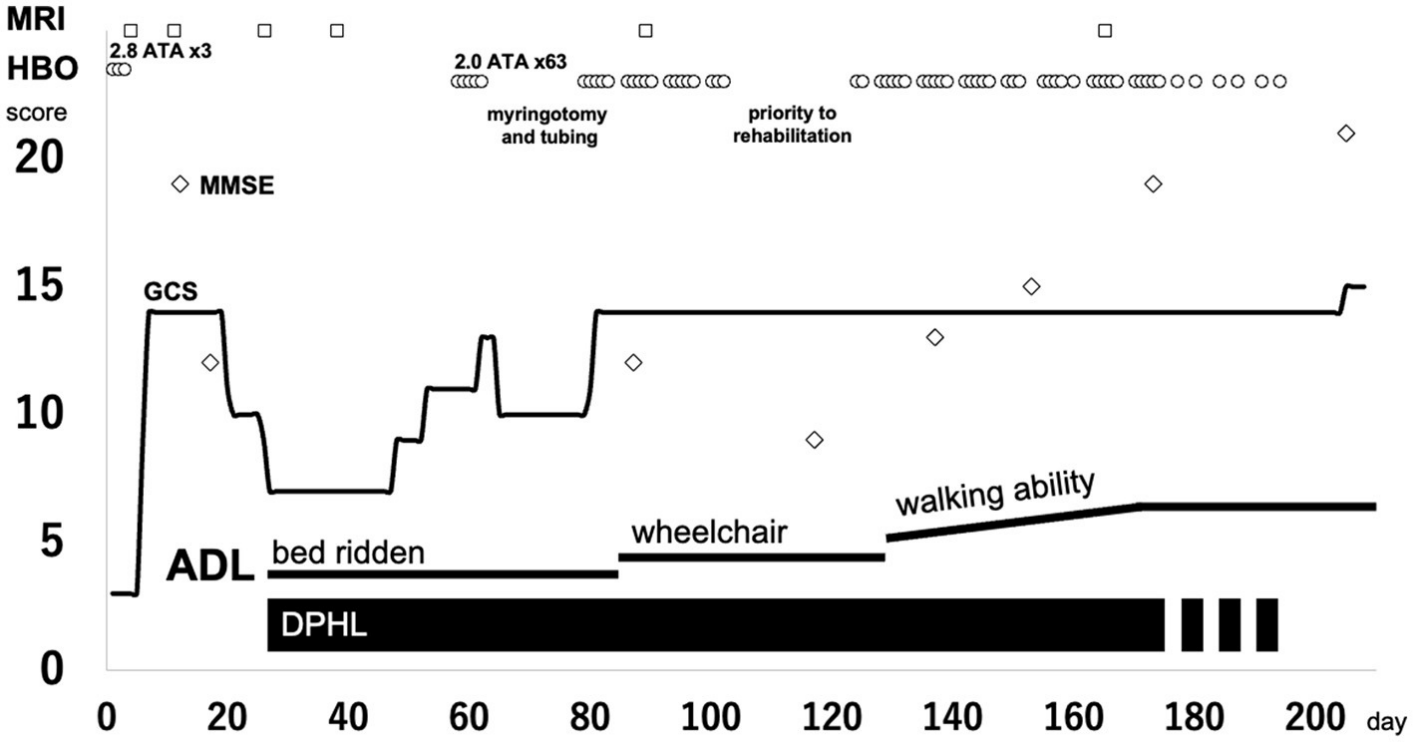
Clinical course of the patient. Circle, HBO; square, MRI taken; diamond, MMSE score. ADL, activities of daily living; DPHL, delayed post-hypoxic leukoencephalopathy; GCS, Glasgow Coma Scale; HBO, hyperbaric oxygen; MMSE, Mini-Mental State Examination; MRI, magnetic resonance imaging.

**Figure 3:**
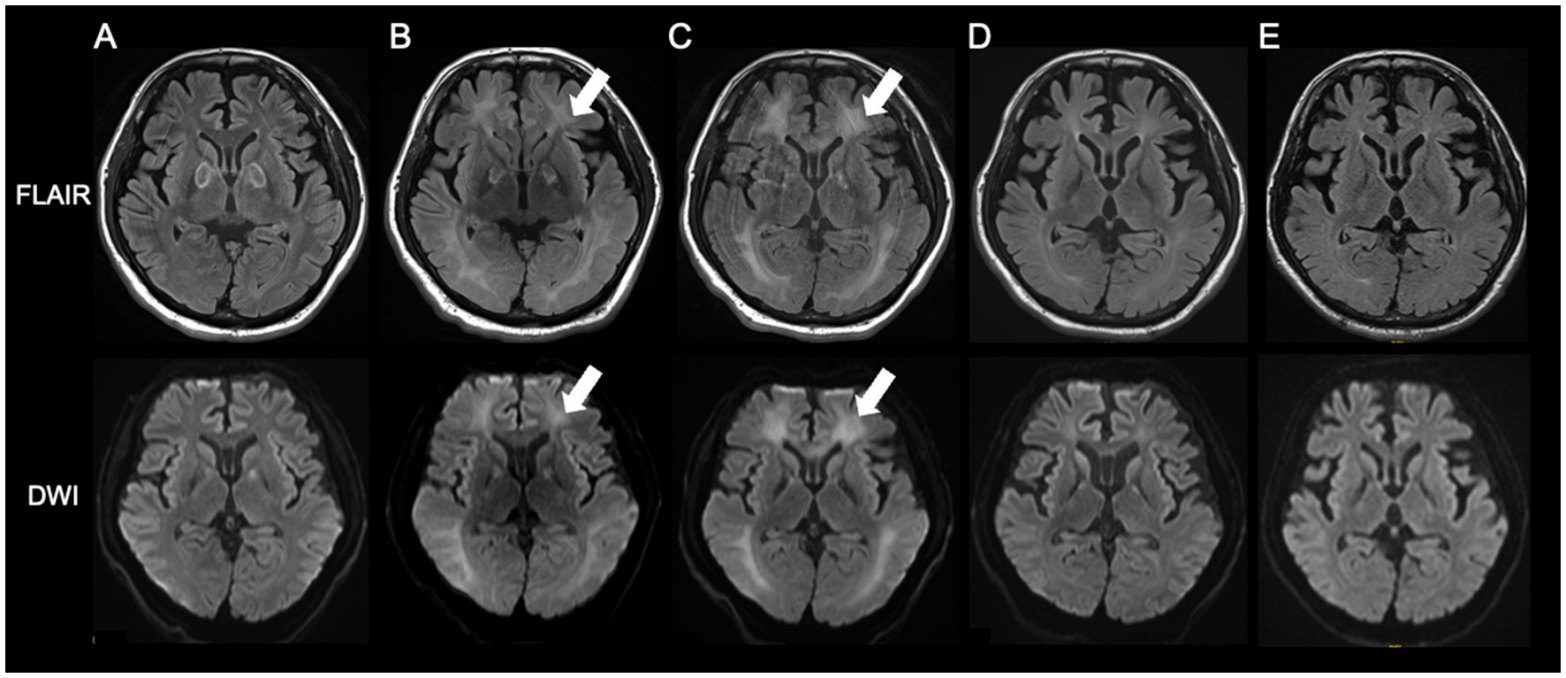
The course of MRI findings. **(A)** day 11; **(B)** day 25; **(C)** day 38; **(D)** day 89; **(E)** day 165. White matter abnormalities become apparent from day 25 and gradually disappear after consecutive HBO therapy. HBO, hyperbaric oxygen; MRI, magnetic resonance imaging.

**Figure 4:**
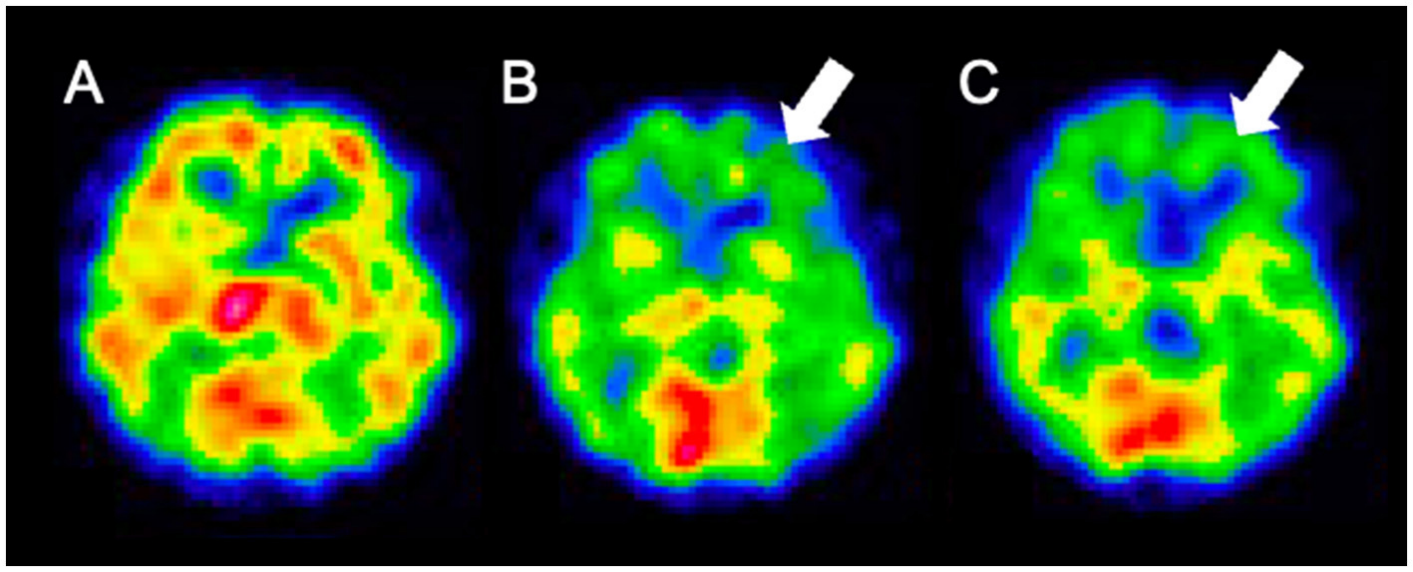
^123^I-IMP SPECT findings. **(A)** day 12; **(B)** day 96; **(C)** day 164. The frontal lobe hypoperfusion is apparent on day 96 and persists. ^123^I-IMP SPECT, N-isopropyl-p-[123I]- iodoamphetamine single-photon emission computed tomography.

Weekday HBO (2.0 ATA, 60 min) trials were started on day 58, regarding the routine HBO regimen for DPHL due to CO poisoning. The patient started to speak at the seventh and showed fidgeting at the ninth HBO session; daily activity gradually improved after that. He was able to spend time in a wheelchair and underwent routine HBO with other patients in a multi-person chamber. At the 40^th^ HBO session, his Mini-Mental State Examination (MMSE) score recovered from an unmeasurable level to 15; the patient was able to walk independently. Although HBO was sometimes interrupted, the patient received 63 HBO treatments (Figure 2; Supplementary Video). The patient eventually reintegrated into society. After initiating HBO, MRI revealed a gradual disappearance of white matter abnormality (Figure 3). However, hypoperfusion in the frontal lobe detected on N-isopropyl-p-[^123^I]-iodoamphetamine single-photon emission computed tomography (^123^I-IMP-SPECT) that appeared during the course, remained evident (Figure 4).

## 3 Discussion

We performed consecutive HBO therapy on the patient, leading to the successful resolution of the serious neurological symptoms. However, the effectiveness of HBO therapy in managing opioid-related DPHL remains uncertain.

We present a patient with tramadol intoxication in the initial phase, whose imaging findings of the affected basal nuclei and cerebellum suggested CHANTER syndrome. However, not all the reported features were present. In addition to naloxone administration and systemic management, we performed HBO therapy, which may have exerted therapeutic effects in the acute phase. However, neurological symptoms recurred, and white matter lesions were observed on MRI in the chronic phase. Furthermore, cerebrospinal fluid analysis suggested that myelin was significantly damaged. The patient's condition gradually deteriorated. However, there were no treatment guidelines for opioid-related DPHL, which made deciding on a treatment plan challenging.

Patients with acute CO poisoning present with impaired consciousness; severe cases present with the involvement of the globus pallidus on MRI. Among these cases, some patients experience DPHL for approximately one month (4). DPHL due to CO poisoning is classically termed delayed neurological sequelae (5). The physiological mechanism of DPHL may involve myelin damage in the white matter. The hypothesis proposing the development of DPHL after a lucid interval is substantiated by factors such as the half-life of myelin (6).

HBO therapy is sometimes performed in patients with DPHL after CO poisoning; some studies have shown its efficacy (7). However, it remains unclear why HBO is effective in DPHL after CO poisoning. Furthermore, the equivalent effectiveness of HBO in DPHL from other DPHL causative agents has never been reported. Based on our experience in treating DPHL due to CO poisoning, a trial of HBO was performed, and significant effectiveness was observed. Concurrent with consecutive HBO therapy, the patient gradually improved the cognitive function and Parkinsonism. However, determining whether HBO was practically effective in this case, whether the patient improved through natural course, or the extent to which HBO was necessary was difficult. Therefore, to verify the effectiveness of HBO, we discontinued the treatment after the 23^rd^ HBO session, allowing the patient to concentrate on rehabilitation. However, the patient's symptoms stopped improving temporarily. Therefore, we resumed HBO after a while. HBO therapy was continued until recovery was validated using imaging studies.

White matter abnormalities observed on MRI gradually disappeared over time. The end of HBO therapy was determined by confirming significant improvement in the white matter lesions on MRI. However, follow-up ^123^I-IMP-SPECT revealed residual damage in the frontal lobe. Severe DPHL cases often involve persistent frontal lobe dysfunction (2). The anatomically longer neuronal tracts in the frontal lobe may render it more susceptible to damage. Clinically, the patient remained incapacitated, reflecting persistent frontal brain dysfunction.

The value of using HBO for the treatment of mental illness has not been well-established (8). For example, the effect of HBO on post-traumatic stress disorder is controversial, with some negative (9) and some positive findings in the literature (10). However, recently, HBO has been reported to be effective in patients with post-stroke depression (11). Since there are some special cases, such as this case, continued research is needed to determine the neuroprotective effects of HBO under certain unique circumstances.

Unfortunately, neither DPHL nor CHANTER syndrome have been elucidated in detail due to their extreme rarity. Furthermore, the limited number of HBO facilities and its specialists has limited the discussion on these conditions. Based on our report, we recommend conducting further studies to confirm the effects of HBO treatment, as our report suggests a possible therapeutic effect of HBO on treating DPHL associated with opioid intoxicated CHANTER syndrome.

In conclusion, the therapeutic effect of HBO is expected in patients with DPHL, even if the cause is not CO poisoning, as HBO enhances myelin regeneration by improving cerebral blood flow and oxygen supply.

## Data availability statement

The original contributions presented in the study are included in the article/Supplementary material, further inquiries can be directed to the corresponding author.

## Ethics statement

Written informed consent was obtained from the individual(s) for the publication of any potentially identifiable images or data included in this article.

## Author contributions

NJ: Conceptualization, Data curation, Formal analysis, Funding acquisition, Visualization, Writing – original draft, Writing – review & editing. KC: Data curation, Writing – review & editing. TN: Visualization, Writing – review & editing. MT: Data curation, Writing – review & editing. KK: Data curation, Writing – review & editing. RT: Data curation, Writing – review & editing. NS: Writing – review & editing, Data curation. SO: Supervision, Writing – review & editing.

## References

[1] Zamora CA Nauen D Hynecek R Ilica AT Izbudak I Sair HI. Delayed posthypoxic leukoencephalopathy: a case series and review of the literature. Brain Behav. (2015) 5:e00364. 10.1002/brb3.364 26357591 PMC4559021

[2] Shprecher D Mehta L. The syndrome of delayed post-hypoxic leukoencephalopathy. NeuroRehabilitation. (2010) 26:65–72. 10.3233/NRE-2010-0536 20166270 PMC2835522

[3] Jasne AS Alsherbini KH Smith MS Pandhi A Vagal A Kanter D. Cerebellar hippocampal and basal nuclei transient edema with restricted diffusion (CHANTER) syndrome. Neurocrit Care. (2019) 31:288–96. 10.1007/s12028-018-00666-4 30788708 PMC6757017

[4] Choi IS. Delayed neurologic sequelae in carbon monoxide intoxication. Arch Neurol. (1983) 40:433–5. 10.1001/archneur.1983.04050070063016 6860181

[5] Myers RA Snyder SK Emhoff TA. Subacute sequelae of carbon monoxide poisoning. Ann Emerg Med. (1985) 14:1163–7. 10.1016/S0196-0644(85)81022-2 4061987

[6] Meyer MA. Delayed post-hypoxic leukoencephalopathy: case report with a review of disease pathophysiology. Neurol Int. (2013) 5:e13. 10.4081/ni.2013.e13 24147210 PMC3794448

[7] Martani L Giovanniello A Bosco G Cantadori L Calissi F Furfaro D. Delayed neurological sequelae successfully treated with adjuvant, prolonged hyperbaric oxygen therapy: review and case report. Int J Environ Res Public Health. (2022) 19:5300. 10.3390/ijerph19095300 35564694 PMC9104642

[8] Hyperbaric Oxygen Therapy for Adults With Mental Illness: a Review of the Clinical Effectiveness. Rapid Response Report: Summary With Critical Appraisal. Ottawa, ON: Canadian Agency for Drugs and Technologies in Health (2014). 25411676

[9] Miller RS Weaver LK Bahraini N Churchill S Price RC Skiba V. Effects of hyperbaric oxygen on symptoms and quality of life among service members with persistent postconcussion symptoms: a randomized clinical trial. JAMA Intern Med. (2015) 175:43–52. 10.1001/jamainternmed.2014.5479 25401463

[10] Doenyas-Barak K Catalogna M Kutz I Levi G Hadanny A Tal S. Hyperbaric oxygen therapy improves symptoms, brain's microstructure and functionality in veterans with treatment resistant post-traumatic stress disorder: a prospective, randomized, controlled trial. PLoS One. (2022) 17:e0264161. 10.1371/journal.pone.0264161 35192645 PMC8863239

[11] Liang XX Hao YG Duan XM Han XL Cai XX. Hyperbaric oxygen therapy for post-stroke depression: a systematic review and meta-analysis. Clin Neurol Neurosurg. (2020) 195:105910. 10.1016/j.clineuro.2020.105910 32474256

